# Supplementary material for: Escherichia coli FtsA forms lipid-bound minirings that antagonize lateral interactions between FtsZ protofilaments
Source: Nat Commun. 2017 Jul 11;8:15957. doi: 10.1038/ncomms15957 (PMC5508204; doi:10.1038/ncomms15957)
Supplement: Supplementary Information [file ncomms15957-s1.pdf]

File name: Supplementary Information
Description: Supplementary Figures, Supplementary Table and Supplementary References

File name: Supplementary Movie 1
Description: Animation of successive tomographic slices through a lipid monolayer with FtsA minirings and FtsZ protofilaments.

File name: Supplementary Movie 2
Description: Animation of the docking of FtsA and FtsZ C-terminal peptide structures as illustrated in Fig. 5h in greater detail.

File name: Peer Review File
Description:

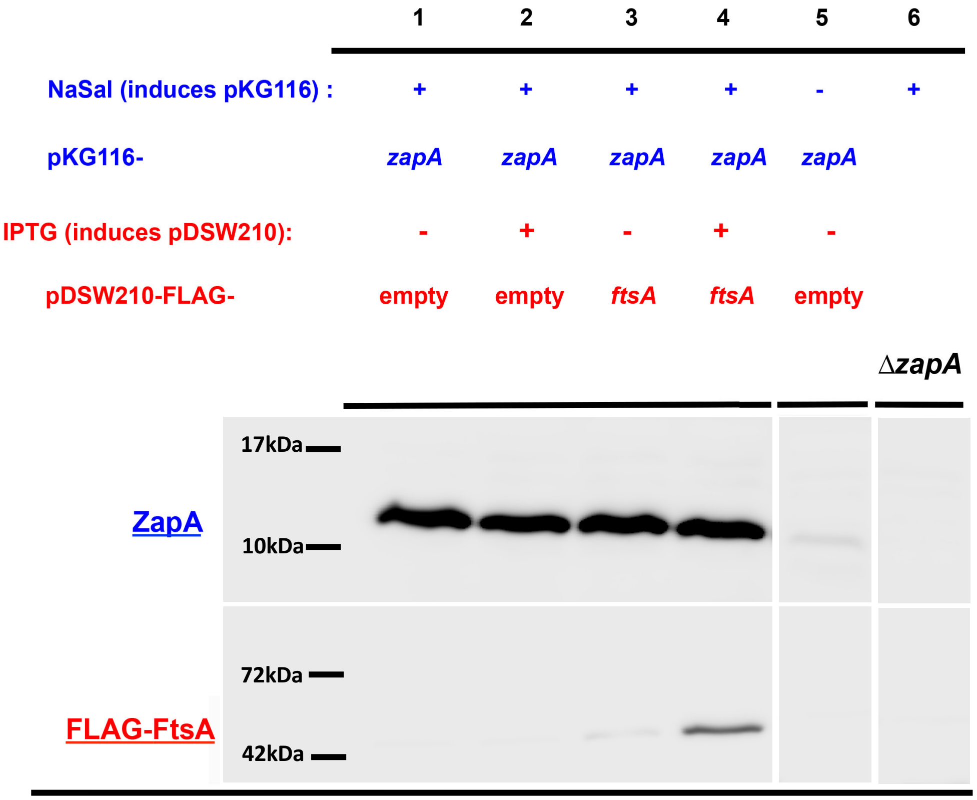

Supplementary Figure 1. Cells co-overproducing ZapA from salicylate-inducible pKG116 and FLAG-FtsA from IPTG-inducible pDSW210F (shown in Fig. 1) have similar excess levels of of ZapA and appropriate increases in FLAG-FtsA levels. Identical blots were probed with anti-ZapA and anti-FLAG (lanes 1-6) confirming that cellular levels of the 12.6 kDa ZapA protein do not change when FLAG-FtsA (46 kDa) is overproduced to suppress the ZapA overproduction phenotype, under conditions shown in Fig. 1a. Without salicylate induction, low levels of ZapA can be detected in lane 5, and no ZapA can be detected in the Δ*zapA* control strain (lane 6). The original blots used to make this figure are shown in Supplementary Fig. 12a.

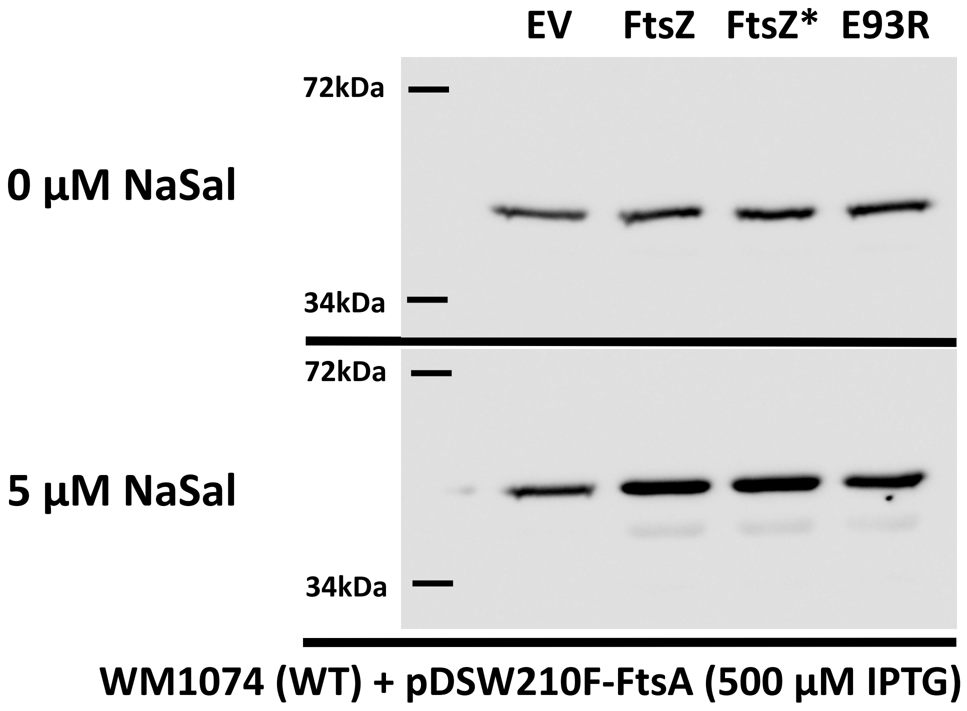

Supplementary Figure 2. Levels of FtsZ from FtsZ bundling mutants are equivalent under uninduced and induced conditions. Cells containing pDSW210F-FtsA and pKG110 derivatives encoding the FtsZ proteins listed at the top were grown with 500 µM IPTG to induce FtsA and either 0 µM sodium salicylate or 5 µM sodium salicylate as in the last column of Fig. 1b. After normalizing for cell density, total cellular proteins were separated by SDS-PAGE, and immunoblotted with anti-FtsZ. The empty vector (EV) lane shows native levels of FtsZ. The original blot used to make this figure is shown in Supplementary Fig. 12b.

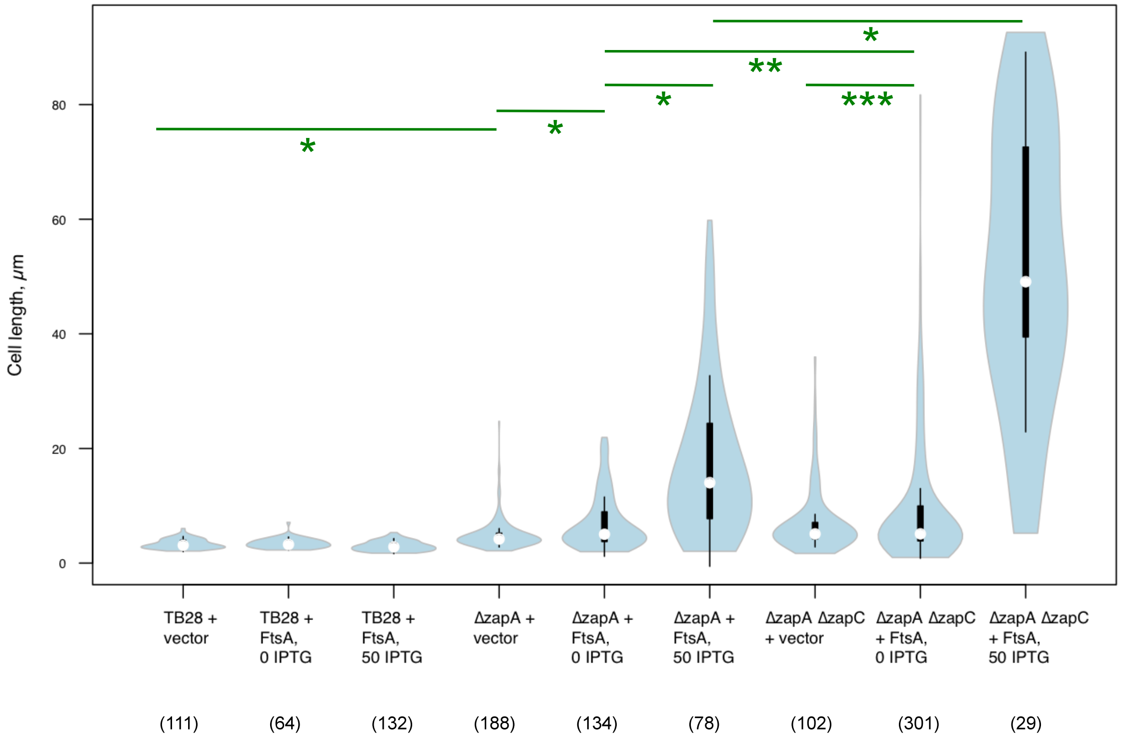

Supplementary Figure 3. The effects of excess FtsA on cell length of TB28 (WT), Δ*zapA*, or Δ*zapA* Δ*zapC* cells. Cells either producing no extra FtsA (vector) or FtsA at uninduced (0 μM IPTG) or moderately induced (50 μM IPTG) levels were assayed for immunofluorescence as in Fig. 2, and their lengths were measured with Oufti software and compiled in Microsoft Excel. Violin plots were made using the BoxPlotR web tool (http://shiny.chemgrid.org/boxplotr/). White boxes lines show the medians; black rectangle limits indicate the 25th and 75th percentiles; whiskers extend 1.5 times the interquartile range from the 25th and 75th percentiles. The blue areas extend vertically to include all outliers; the widths of the blue areas represent the probability densities. The number of cells counted for each condition is shown in parentheses. Selected unpaired two-sample t-tests are shown by the green lines and asterisks, with * denoting $p < 0.0001$; ** denoting $p < 0.001$; *** denoting $p < 0.01$.

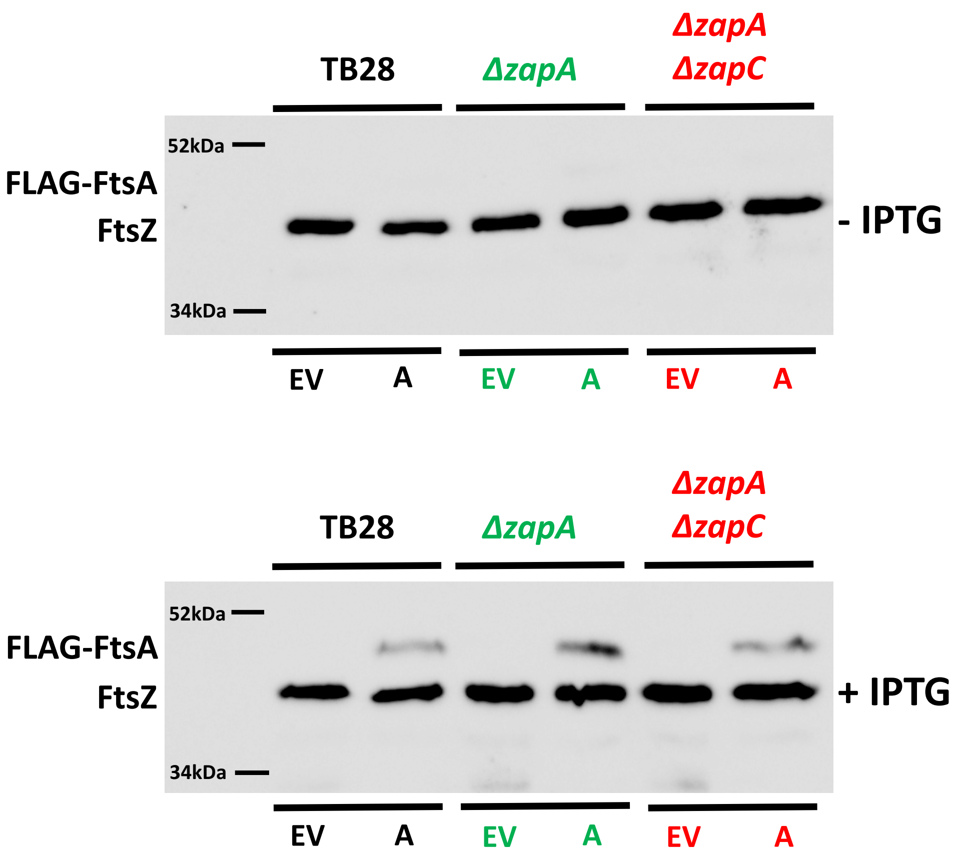

Supplementary Figure 4. Levels of endogenous FtsZ or FLAG-FtsA from pDSW210F plasmids ± IPTG are similar among wild-type (TB28), Δ*zapA* or Δ*zapA* Δ*zapC* cells. Cells carrying the plasmid constructs listed at the bottom (EV, empty vector; A, FtsA) were grown and induced with 50 μM IPTG as in Fig. 2. After normalizing for cell density, total cell proteins were separated by SDS-PAGE and immunoblots were probed with α-FtsZ to detect endogenous FtsZ (40 kDa) or α-FLAG to detect FLAG-FtsA (46 kDa). The original blot used to make this figure is shown in Supplementary Fig. 12c.

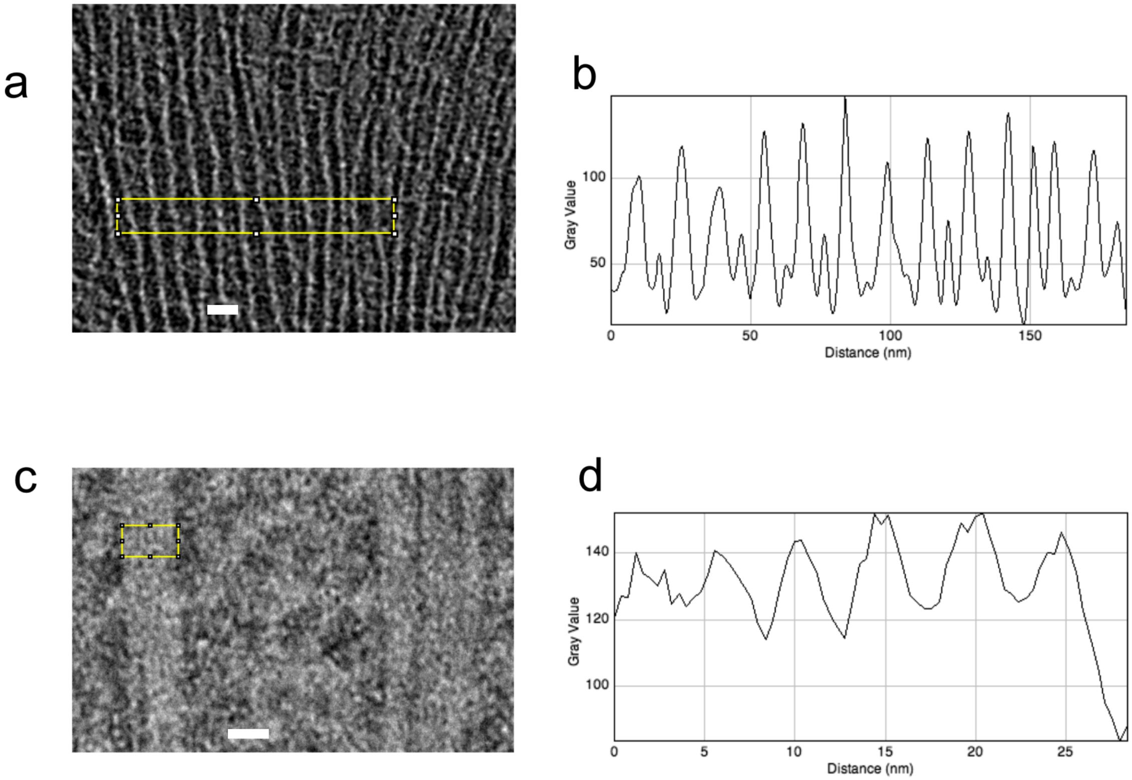

Supplementary Figure 5. Comparison between aligned but unbundled FtsZ protofilaments and strongly bundled FtsZ* protofilaments, assembled on lipid monolayers with FtsA*. (a-b): tomogram showing the 14-15 nm periodicity between filaments of FtsZ (5 μM) on top of minirings of FtsA (0.5 μM); (c-d), 0.5 μM FtsA* + 5 μM FtsZ* bundle showing the 5 nm periodicity between protofilaments. Intensity plots of the boxed regions shown were analyzed in ImageJ. Scale bars = 20 nm.

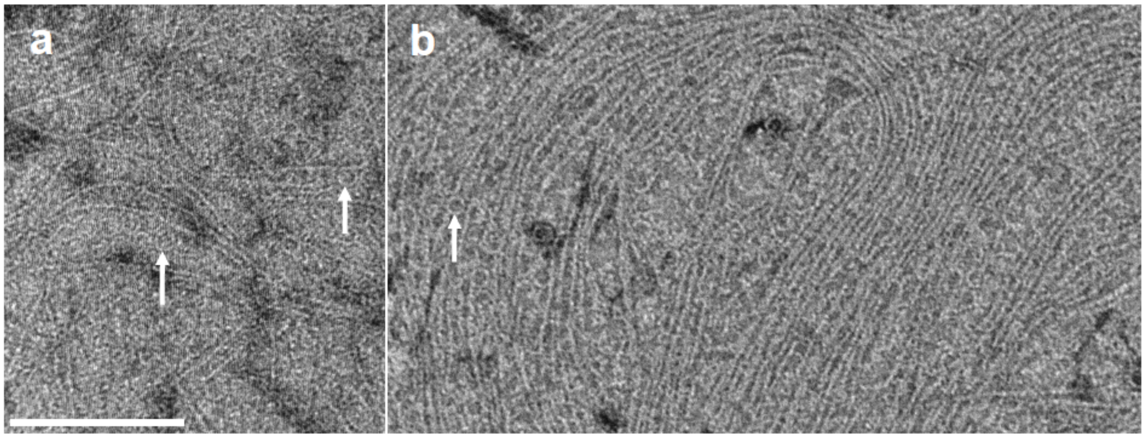

Supplementary Figure 6. Time-lapse assembly of FtsZ polymers on FtsA minirings. FtsZ was polymerized with GTP on the pre-assembled FtsA minirings as described in Methods. Grids were negatively stained 30 seconds (a) and 5 minutes (b) after GTP addition. Arrows point to one of many FtsA minirings present. Scale bar, 200 nm.

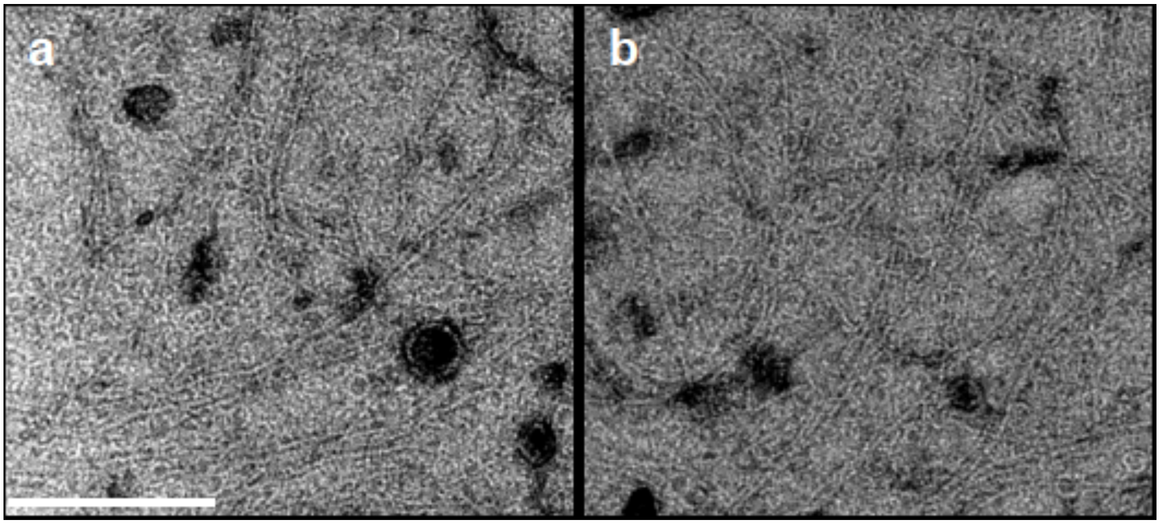

Supplementary Figure 7. **Simultaneous addition of FtsA and FtsZ to lipid monolayers displays patterns similar to those from sequential addition of FtsA and FtsZ**. ATP was included in the mixture of FtsA (0.5 μM) + FtsZ (2.5 μM). FtsZ polymerization was triggered by addition of GTP and the mixture was incubated for 20 minutes before removing the grid. Two representative photos are shown. Scale bar, 200 nm.

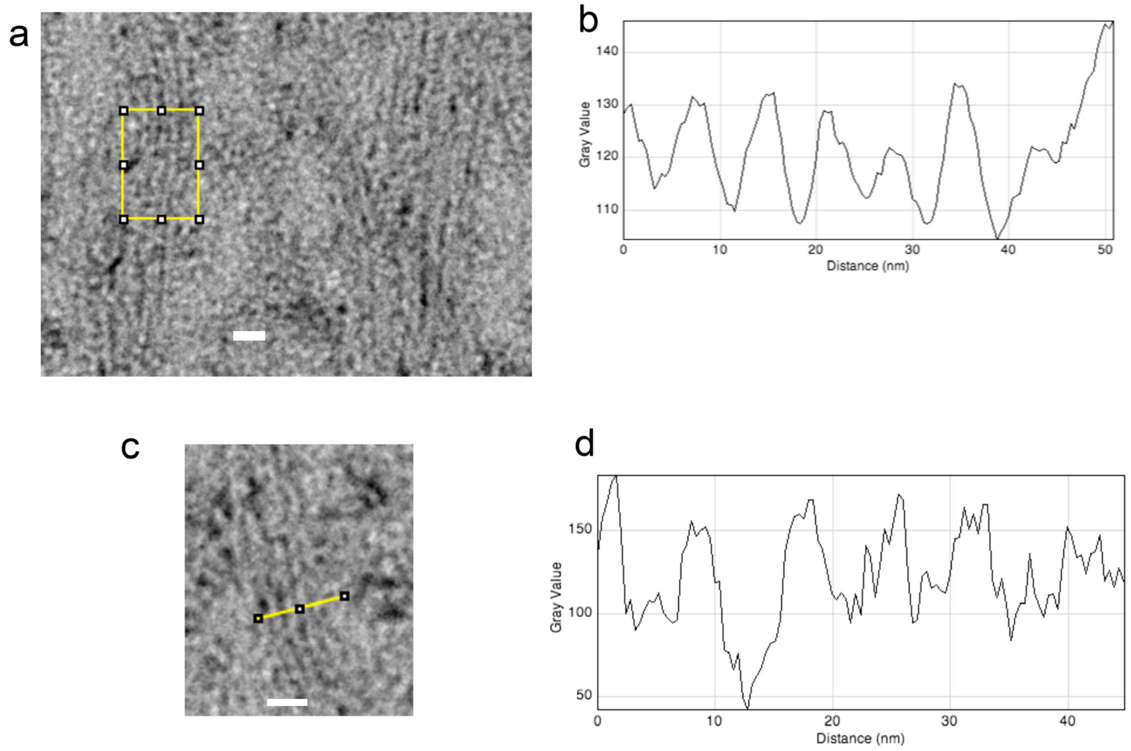

Supplementary Figure 8. Measurements of FtsZ (5 μM) protofilament bundles when assembled on lipid monolayers seeded with 0.5 μM FtsA*. (a-b) TEM showing 7-8 nm periodicity between filaments in a bundle. (c-d) Another bundle with mostly similar spacing. Intensity plots of the boxed regions or line shown were analyzed in ImageJ. Scale bars = 20 nm.

a b

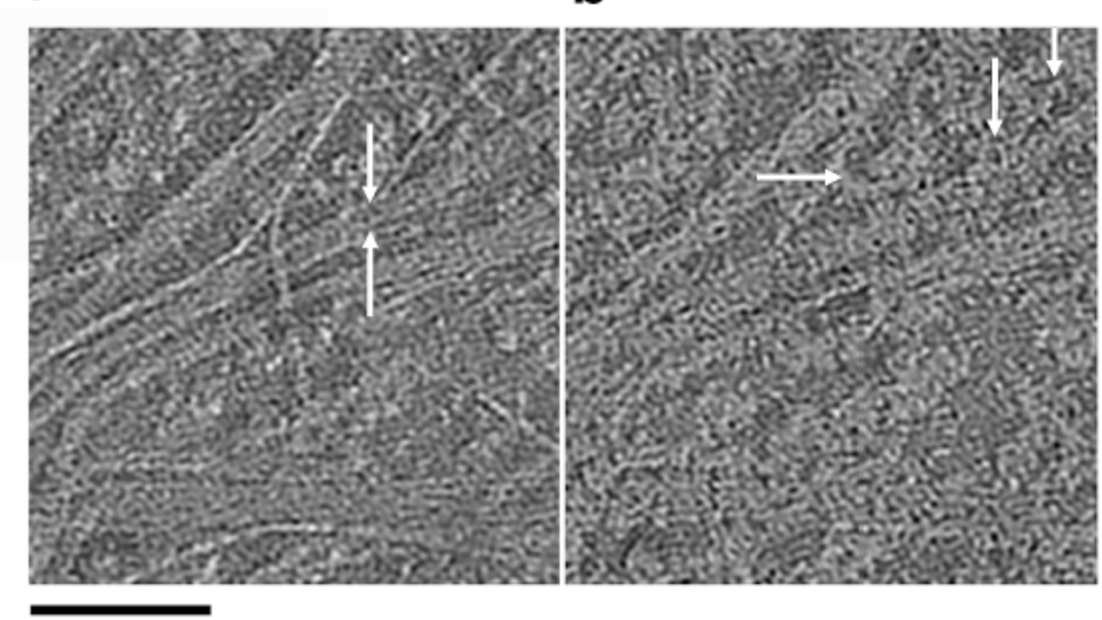

Supplementary Figure 9. Behavior of FtsA* and WT FtsZ on lipid monolayers. A membrane distal (a) or proximal (b) pair of tomographic slices of a TEM image of a lipid monolayer seeded with 0.5 μM FtsA*, then overlaid with 2.5 μM FtsZ under standard assay conditions, showing FtsZ protofilament bundles (arrows in a) or FtsA* oligomeric structures (arrows in b). Scale bar, 100 nm.

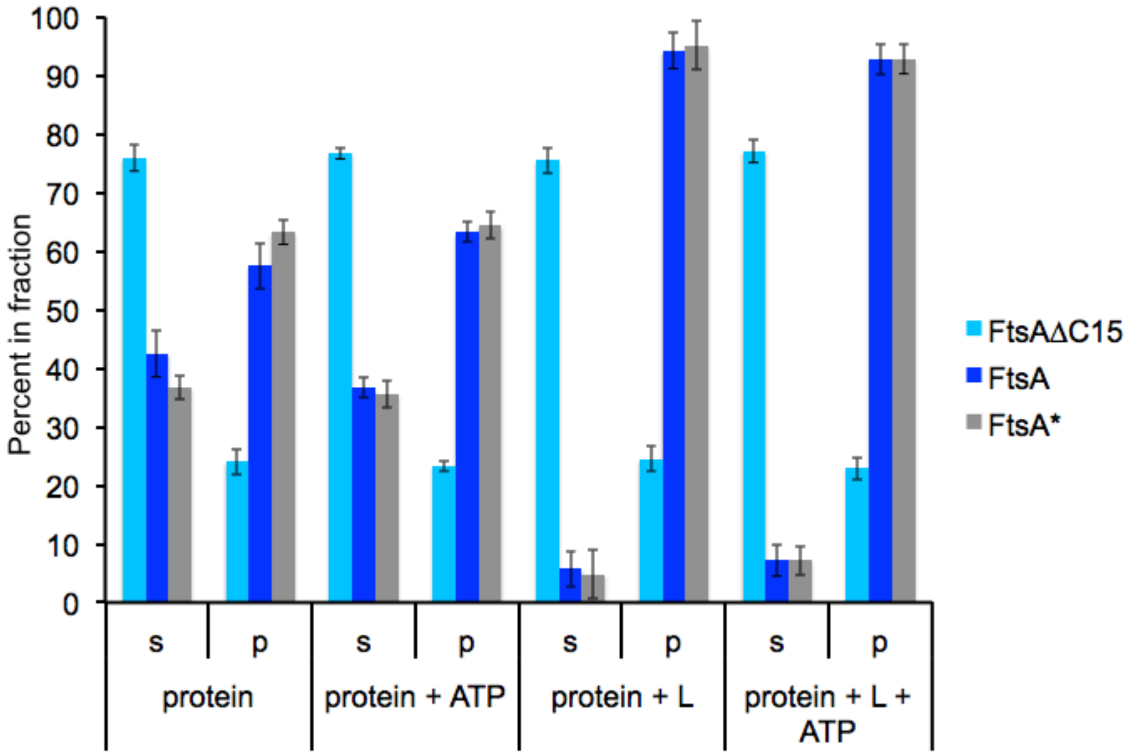

Supplementary Figure 10. FtsA and FtsA* bind lipids equally well. Shown are the results of liposome sedimentation experiments with purified FtsA, FtsA* or the membrane binding-defective FtsAΔC15. Protein pellet (p) and supernatant (s) fractions after sedimentation with or without lipids (L) or added ATP were separated by SDS-PAGE and stained with Coomassie Blue. The densities of the protein bands from three independent experiments were quantitated using ImageJ [1]; error bars represent standard deviations.

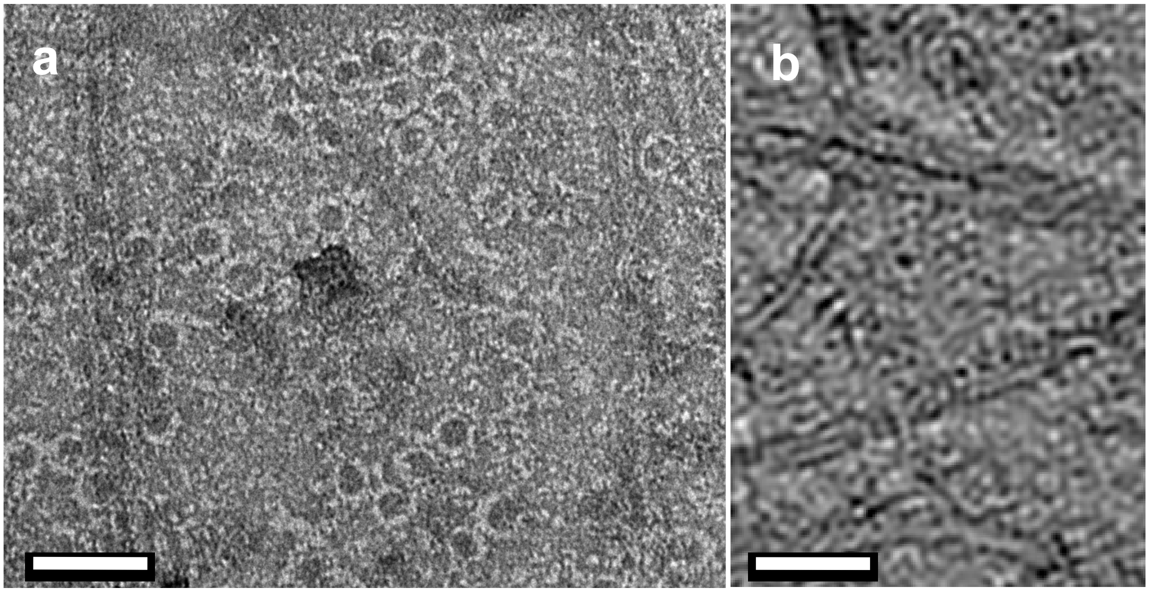

Supplementary Figure 11. Behavior of FtsZ* and WT FtsA on lipid monolayers. (a) Standard TEM image of a lipid monolayer seeded with 0.5 μM FtsA + 1 μM FtsZ* under standard assay conditions, showing FtsA minirings and FtsZ* protofilament pairs. No FtsA minirings associate directly with FtsZ* protofilaments in this or other images under these conditions. Scale bar, 50 nm. (b) A tomographic slice of a lipid monolayer seeded with 0.5 μM FtsA and 2.5 μM FtsZ* under standard assay conditions, showing lack of FtsA minirings, although many curved oligomers are visible. Scale bars, 40 nm.

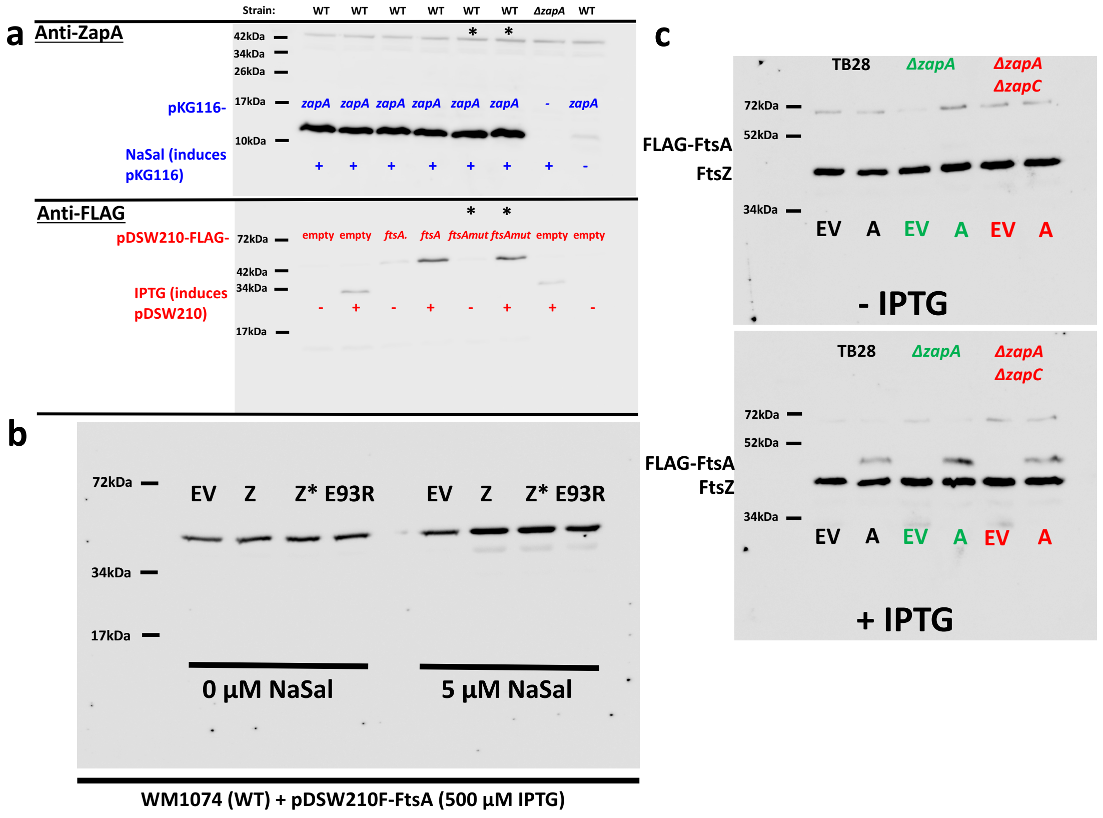

Supplementary Figure 12. Original full immunoblots. Portions of these blots were used to make Supplementary Figures 1 (a), 2 (b), and 4 (c), respectively. Labeling is described in the respective figure legends, except that lanes in (a) marked with an asterisk correspond to a mutant FtsA were not used for this study.

Supplementary Table 1.

Plasmid	Description	Source/reference
pKG110	pACYC184 derivative with *nahG* promoter	[2]
pDH156	pKG110-*ftsZ*$_{WT}$	[3]
pDH155	pKG110-*ftsZ**	[3]
pWM5082	pKG110-*ftsZ*$_{E93R}$	Daniel Haeusser
pKG116	pKG110 with stronger ribosome binding site	[4]
pWM4651	pKG116-*zapA*	This study
pDSW210F	*colE1* plasmid with weakened P_{trc} promoter and N-terminal *flag* epitope	[5]
pWM2785	pDSW210F-*ftsA*$_{WT}$	[5]
pWM3169	pDSW210F-*ftsA*$_{W408E}$	[6]
pWM3171	pDSW210F-*ftsAΔC15*	[6]
pWM971	*ftsZ* in pET11a vector	[7]
pDH160	*ftsZ** in pET11a vector	[3]
pWM1260	*his*$_6$-*ftsA* in pET28a vector	[8]
pWM1609	*his*$_6$-*ftsA** in pET28a vector	[8]
pWM4908	*his*$_6$-*ftsAΔC15* in pET28a vector	This study

Supplementary References

1. Schneider, C. A., Rasband, W. S. & Eliceiri, K. W. NIH Image to ImageJ: 25 years of image analysis. *Nat. Methods* **9,** 671–675 (2012).
2. Mowery, P., Ames, P., Reiser, R. H. & Parkinson, J. S. Chemotactic signaling by single-chain chemoreceptors. *PLoS One* **10,** e0145267 (2015).
3. Haeusser, D. P., Rowlett, V. W. & Margolin, W. A mutation in *Escherichia coli* ftsZ bypasses the requirement for the essential division gene *zipA* and confers resistance to FtsZ assembly inhibitors by stabilizing protofilament bundling. *Mol. Microbiol.* **97,** 988–1005 (2015).
4. Han, X.-S. & Parkinson, J. S. An unorthodox sensory adaptation site in the *Escherichia coli* serine chemoreceptor. *J. Bacteriol.* **196,** 641–649 (2014).
5. Shiomi, D. & Margolin, W. Dimerization or oligomerization of the actin-like FtsA protein enhances the integrity of the cytokinetic Z ring. *Mol. Microbiol.* **66,** 1396–1415 (2007).
6. Shiomi, D. & Margolin, W. Compensation for the loss of the conserved membrane targeting sequence of FtsA provides new insights into its function. *Mol. Microbiol.* **67,** 558–569 (2008).
7. Bramhill, D. & Thompson, C. M. GTP-dependent polymerization of *Escherichia coli* FtsZ protein to form tubules. *Proc. Natl. Acad. Sci. USA* **91,** 5813–5817 (1994).
8. Geissler, B., Elraheb, D. & Margolin, W. A gain of function mutation in *ftsA* bypasses the requirement for the essential cell division gene *zipA* in *Escherichia coli*. *Proc. Natl. Acad. Sci. USA* **100,** 4197–4202 (2003).
